# Regulator of G-Protein Signaling – 5 (RGS5) Is a Novel Repressor of Hedgehog Signaling

**DOI:** 10.1371/journal.pone.0061421

**Published:** 2013-04-18

**Authors:** William M. Mahoney, Jagadambika Gunaje, Guenter Daum, Xiu Rong Dong, Mark W. Majesky

**Affiliations:** 1 Department of Pathology, University of Washington, Seattle, Washington, United States of America; 2 Department of Surgery, University of Washington, Seattle, Washington, United States of America; 3 Department of Pediatrics, University of Washington, Seattle, Washington, United States of America; 4 Center for Cardiovascular Biology, University of Washington, Seattle, Washington, United States of America; 5 Institute for Stem Cell and Regenerative Medicine, University of Washington, Seattle, Washington, United States of America; 6 Seattle Children’s Research Institute, University of Washington, Seattle, Washington, United States of America; Medical School of Hannover, United States of America

## Abstract

Hedgehog (Hh) signaling plays fundamental roles in morphogenesis, tissue repair, and human disease. Initiation of Hh signaling is controlled by the interaction of two multipass membrane proteins, patched (Ptc) and smoothened (Smo). Recent studies identify Smo as a G-protein coupled receptor (GPCR)-like protein that signals through large G-protein complexes which contain the Gα_i_ subunit. We hypothesize Regulator of G-Protein Signaling (RGS) proteins, and specifically RGS5, are endogenous repressors of Hh signaling via their ability to act as GTPase activating proteins (GAPs) for GTP-bound Gα_i_, downstream of Smo. In support of this hypothesis, we demonstrate that RGS5 over-expression inhibits sonic hedgehog (Shh)-mediated signaling and osteogenesis in C3H10T1/2 cells. Conversely, signaling is potentiated by siRNA-mediated knock-down of RGS5 expression, but not RGS4 expression. Furthermore, using immuohistochemical analysis and co-immunoprecipitation (Co-IP), we demonstrate that RGS5 is present with Smo in primary cilia. This organelle is required for canonical Hh signaling in mammalian cells, and RGS5 is found in a physical complex with Smo in these cells. We therefore conclude that RGS5 is an endogenous regulator of Hh-mediated signaling and that RGS proteins are potential targets for novel therapeutics in Hh-mediated diseases.

## Introduction

Hh signaling is an important mediator of cell proliferation, morphogenesis, and wound repair, and it plays critical roles in organogenesis, tissue fibrosis, and different forms of cancer [1]–[4]. Shh has been reported to stimulate angiogenesis [5], [6], exhibit anti-inflammatory properties [7], and maintain various stem and progenitor cell populations via its mitogenic and survival activity for these cells [8]–[10]. Despite the importance for normal development and tissue homeostatsis, a complete understanding of how Hh proteins signal in mammalian cells is still lacking. This is particularly true with regard to endogenous regulatory pathways that inhibit, rather than stimulate Hh signaling.

Genetic and biochemical evidence has shown that Smo, a seven transmembrane domain protein with structural homology to GPCRs, initiates Hh signaling in Hh responsive cell types [11]–[14]. GPCRs are among the most abundant gene families in the mammalian genome (∼1% of all coding genes) [12], and are frequent pharmaceutical targets [15], [16]. In the absence of agonist, the 3^RD^ intercellular loop (i3) of a GPCR interacts with the large G proteins: a GDP-bound Gα protein (Gα_s_, Gα_q_, Gα_i/o_, and/or Gα_12/13_) and the Gβγ heterodimer. Upon agonist binding, GTP is exchanged with GDP on the Gα protein, which then dissociates from the Gβγ subunits and activates down-stream signaling thorough secondary messengers [17]–[19]. Regulator of G-protein Signaling (RGS) proteins, of which there are more than 20 mammalian family members [20]–[23], function as GAPs that greatly accelerate the GTP hydrolyzing activity of the Gα protein; the GDP-bound Gα subunit is inactive for signaling [24], [25]. In addition to signaling through a GPCR, Smo-mediated signaling is controlled through the coordinated localization of the signaling complex to a unique cell organelle, the primary cilia [1], [26]–[30]. Unlike most GPCRs, Smo-dependent signaling is constitutively active; however, though the localization of Ptc to primary cilia, signaling is inhibited [31], [32]. In the presence of Shh, which binds directly to Ptc, Ptc translocates out of the cilia, allowing Smo to enter the cilia and actively signal [33]–[36]. Therefore, signaling through GPCRs is the product of proper cellular localization and specific interactions between the GPCR agonist, the GPCR itself, individual large G proteins, and specific RGS proteins.

Recent studies have identified the Gα proteins which interact with Smo. *In vitro*, Smo is capable of signaling through Gα_i1–3_, Gα_o_, and Gα_z_ [37]. In Drosophila, Smo signals through Gα_i_ [38], as it does in neuronal precursor cells [39]. Given the facts that members of the RGS-R4 subfamily of RGS proteins show specificity for Gα_i_ and Gα_q_, and that RGS5 interacts with Gα_i1–3_ [40], we hypothesize that RGS5 is a component of the Hh signaling cascade that functions to dampen Smo-dependent signaling through its interaction with Gα_i_.

## Materials and Methods

### Cell Culture

C3H10T1/2 cells (ATCC) were cultured in BME (Gibco) with 10% fetal bovine serum (PAA Laboratories) and penicillin-streptomycin. Cells were grown at 37°C and 5% CO_2_. For qPCR analysis, 5.0×10^4^ cells were plated in a 6-well dish. For protein extracts, 1.0×10^6^ cells were plated in a 15 cm plate. Where indicated, cells were treated with Shh (1 µg/mL; R&D Systems) for 2 hours, 6 hours, or 24 hours. Where indicated, cells were treated for 24 hours with SAG (100 nM; Santa Cruz) or pertussis toxin (PTX; 100 ng/mL; List Biological Laboratories Inc.).

### Plasmids

The plasmids encoding the human RGS1, RGS2, RGS3, RGS4, RGS5, and RGS16 (3X HA-tagged, N-terminus) were obtained from the Guthrie cDNA Resource Center. The HA epitope tags were removed and a FLAG epitope tag was added to either the N- or C-terminus of RGS5 by standard cloning techniques. The following primers were used to add a FLAG epitope tag to the N-terminus of hRGS5 (referred to as FL-hRGS5; sequence encoding FLAG tag is underlined): 5′- AACTTTAAGCTTATGGCAGATTATAAAGATGATGATGATAAATGCAAAGGACTTGCAGCTTTGCCC CAC-3′; 5′-CAGGAGTTAATCAAGTAGCTCGAGTCTAGAGGGCCCGTTTA-3′. The following primers were used to add a FLAG epitope tag to the C-terminus of hRGS5 (referred to as hRGS5-FL; sequence encoding FLAG tag is underlined): 5′-GCTAGCGTTTAAACTTAAGCTTGGTACCACCATGTGCAAAGGACTT-3′; 5′-GAGTTTTATCAGGAGTTAATCAAGGATTATAAAGATGATGATGATAAATAACTCGAGCCCCGC-3′. The resulting plasmids were sequence verified and the size of the resultant protein was confirmed by immunonblot.

### Quantitative Real-time RT-PCR (qPCR) and Data Analysis

RNA was isolated from C3H10T1/2 cells using the RNeasy RNA isolation mini kit (Qiagen) as described in Gunaje *et al* [41]. The mouse genes assayed via TaqMan probes were the following: RGS5 (Mm00501393_m1; Applied Biosystems), RGS2 (Mm00501385_m1; Applied Biosystems), RGS4 (Mm00501389_m1; Applied Biosystems), Smo (Mm01162710_m1; Applied Biosystems), Ptc1 (Mm00436026_m1; Applied Biosystems), Ptc2 (Mm00436047_m1; Applied Biosystems), Gli1 (Mm00494645_m1; Applied Biosystems), Gli2 (Mm01293116_m1; Applied Biosystems), and GAPDH (Mm99999915_m1; Applied Biosystems). The mouse genes assayed via SYBR-Green probes were the following: collagen, type 1, alpha 1 (Col1α1; 5′-ATGATGCTAACGTGGTTCGT-3′, 5′-TGGTTAGGGTCGATCCAGTA-3′), bone sialoprotein (Bsp; 5′-AGAACAATCCGTGCCACTCACT-3′, 5′-CCCTGGACTGGAAACCGTTT-3′), osterix (Osx; 5′-AGAGATCTGAGCTGGGTAGAGGAA-3′, 5′-AAGTTGAGGAGGTCGGAGCAT-3′), related transcription factor 2 (Runx2; 5′-ATGCCTCCGCTGTTATGAAA-3′, 5′-GAATGCGCCCTAAATCACTGA-3′), and GAPDH (5′-CTGGAGAAACCTGCCCAAGTA-3′, 5′-TGTTGCTGTAGCCGTATTCA-3′). Gene expression was calculated by the ΔΔCt method: Fold expression  = 2^−ΔΔCt^. Specifically, gene expression was corrected for GAPDH expression within each sample and then normalized to an individual treatment condition within each dataset (as indicated in the figure legend).

### siRNA Knockdown of RGS5 Expression

RGS5 was knocked-down in C3H10T1/2 cells using a specific small interfering RNA (siRNA) from Invitrogen ((1) 5′-CAGACUCUGCUGUUGACCUUGUCAU-3′, or (2) 5-GGUGAACAUUGACCACUUCACUAAA-3′ (Fig. S5)). RGS4 was knocked-down using a specific siRNA from Invitrogen (5′-AAAGCUGCCAGUCCACAUUCAUGGU-3′). In control experiments, a non-specific siRNA was utilized (Invitrogen). Cells were transfected with siRNA with Fugene 6 (Roche) following manufacturers specifications. After 24 hours, the cells were changed to serum-free media and starved for 24 hours prior to stimulation with Shh (1 µg/mL) for 2 hours, 6 hours, or 24 hours, where indicated. Alternatively, cells were stimulated with SAG (100 nM) for 24 hours, where indicated.

### Osteogenesis Assay

C3H10T1/2 cells were cultured in the presence or absence of SAG (100 nM) and the presence or absence of over-expressed FL-hRGS5 (375 ng/12-well dish) with Fugene 6 (Roche) following manufacturers specifications. Following 21 days of culture (media was changed every 3–4 days throughout the 3-week period), RNA was isolated and gene expression of multiple markers of bone development was determined as above.

### Immunoflurescence

C3H10T1/2 cells were transfected with the FL-hRGS5 expression vector (600 ng/6-well dish) with Fugene 6 (Roche) following manufacturers specifications. Following 24 hours, cells were fixed with 4% formaldehyde (in PBS with 0.1% Triton X-100) for 5 minutes at room temperature. After washing cells 3 times in PBS with 0.1% Triton X-100, cells were blocked with 1% BSA (in PBS with 0.1% Triton X-100) for 30 minutes. Cells were incubated with primary antibody (either α-acetylated tubulin (Sigma; 2.8 µg/mL) or α-Flag (Sigma; 5 µg/mL)) for 1 hour at room temperature. Following 3 washes with PBS with 0.1% Triton X-100, cells were incubated with secondary antibody (either Alexa Fluor 594 (Invitrogen; 1/1000; for α-acetylated tubulin) or Alexa Fluor 488 (Invitrogen; 1/1000; for α-Flag)) for 1 hour at room temperature. Following 3 washes with PBS with 0.1% Triton X-100, cells were stained with DAPI (1/1000; Sigma) and visualized.

### Co-Immunoprecipitation Assay and Immunoblotting

C3H10T1/2 cells were transfected with expression vectors (3 µg/100 mm plate) encoding human RGS proteins containing either a 5′- or 3′-Flag epitope tag, FL-hRGS5 and hRGS5-FL, respectively, or with a HA epitope tag (HA-RGS1, -RGS2, -RGS3, -RGS4, -RGS5, RGS8 and -RGS16) with Fugene6 (Roche) following manufacturers specifications. Whole cell extracts were prepared by resuspending the cell pellet 24 hours following transfection in cell lysis buffer (50 mM Tris/HCl, pH 8.0; 120 mM NaCl; 0.5% NP-40; 1 mM EDTA). 100 µg of protein extracts were incubated with 1.4 µg of α-acetylated tubulin (Sigma), 1 µg α-Smo (Sigma), or 1 µg β-tubulin antibody (Cell Signaling) overnight at 4°C with rocking. 20 µL of Protein A/G+-agarose (Santa Cruz; for immunoprecipitation of Smo- and β-tubulin) or Protein G+ agarose (Santa Cruz; for immunoprecipitation of acetylated tubulin-bound complexes) was added to the protein extracts and incubated for 4 hours at 4°C with rocking. The beads were washed 3X with 1 mL of lysis buffer, followed by centrifugation at 2500 rpm. The beads were resuspended in 40 µL lysis buffer and 8 µL 6X SDS/PAGE sample buffer. Extracts were boiled and proteins were separated by SDS/PAGE (10% gel) and transferred to a PVDF membrane, which was subsequently blocked with 5% nonfat dry milk (NFDM) in TBS-T (0.1% Tween). Membranes were incubated with the α-FLAG-HRP antibody (1 µg/mL; Sigma) or the α-HA antibody (1/500; Roche Applied Sciences) overnight at 4°C. After 4X washes with TBS-T, membranes were incubated with 1∶15,000 goat α-rat IgG HRP (Jackson Immuno Research). After 4X washes with TBS-T, membranes were incubated with ECL reagent (Super Signal West Pico, Pierce) and exposed to autoradiographic film.

### Statistics

Data is represented as average ± standard error means (SEM) or standard deviation (SD), as indicated in the figure legend. Differences in data were considered if p<0.05, as determined by student’s t-test.

## Results

To investigate the potential function of RGS5 in control of Hh signaling, we determined the effects of RGS5 over-expression on Shh reporter gene expression in C3H10T1/2 cells. C3H10T1/2 cells are a murine embryonic mesenchymal cell line [42] commonly used to study mammalian Hh signaling [43]. These cells were used to develop and evaluate antagonists of Hh-mediated signaling [44]–[46] and to establish the role of Shh and BMP-2 in chrondrogenic and osteogenic differentiation [47]–[50]. We confirmed that similar to other cells [14], [38], [51], Shh signals through a Gα_i_-dependent pathway in C3H10T1/2 cells. Specifically, we demonstrated that Shh reporter gene expression is inhibited in the presence of pertussis toxin (PTX; Fig. S1). Furthermore, C3H10T1/2 cells exhibit properties of a progenitor for vascular smooth muscle cells and pericytes [52] and RGS5 expression is characteristic of pericytes [53]–[55]. In pericytes, Shh signaling coordinates vascular outgrowth in the choroid plexus [56] and promotes blood brain barrier properties in perivascular astrocytes [57]. Thus, RGS5 is expressed in pericytes and mural cells and may play an important role in the regulation of Shh-mediated vascular development and angiogenesis.

### RGS5 Over-expression Inhibits Shh-mediated Signaling

**Figure 1:**
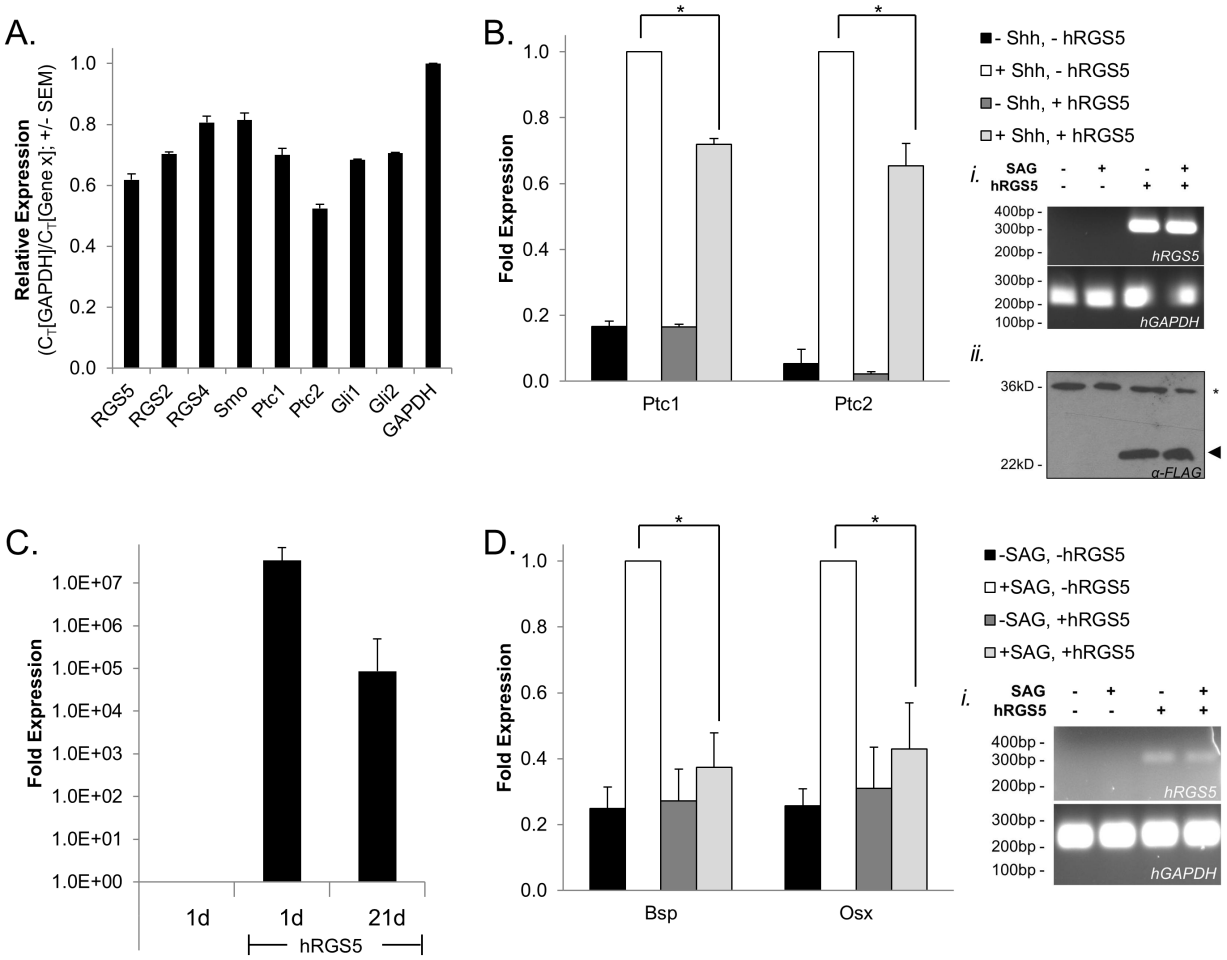
RGS5 inhibits Shh-mediated reporter expression. (**A**) Basal expression level of various members of the RGS-R4 subfamily (RGS5, RGS2, and RGS4) and members of the Shh signaling cascade (Smo, Ptc1, Ptc2, Gli1, and Gli2) in C3H10T1/2 cells. Expression of the indicated genes was determined by qPCR and represented relative to GAPHD expression. (n = 3–7; error bars = SEM) (**B**) Over-expression of RGS5 inhibits Shh-mediated reporter expression. RGS5 was over-expressed in C3H10T1/2 cells by transient transfection and treated with Shh (1 µg/mL) for 24 hours. The effect of RGS5 over-expression on Ptc1 and Ptc2 was assessed by qPCR. Expression is corrected for GAPDH expression and normalized to the expression of each gene following 24hr of Shh treatment, but in the absence of RGS5 over-expression (white bar). (n = 3; error bar = SEM; *p<0.05 by t-test) (**Bi&ii**) Insets: traditional RT-PCR (30 cycles) demonstrating hRGS5 message (**i**) and protein (**ii**) in transfected C3H10T1/2 cells. (**C&D**) Over-expression of RGS5 inhibits Shh-mediated osteogenesis. RGS5 was over-expressed in C3H10T1/2 cells (**C**), and gene expression was monitored over 21 days by qPCR. Expression is corrected for GAPDH expression and normalized to the expression of hRGS5 in untransfected cells. Following 21 days of SAG treatment (100nM), RNA was isolated and the expression of Bsp and Osx was determined by qPCR. Expression is corrected for GAPDH expression and normalized to the expression of each gene following 21 days of SAG treatment, but in the absence of RGS5 over-expression (white bar). (n = 3; error bar = SEM; p<0.05 by t-test) (**Di**) Inset: traditional RT-PCR (30 cycles) demonstrating hRGS5 message expression in transfected C3H10T1/2 cells.

We demonstrate C3H10T1/2 cells express members of the Shh-signaling cascade (Smo, Ptc1, Ptc2, Gli1, and Gli2) and multiple members of the RGS-R4 subfamily (RGS2, RGS4, and RGS5) (Fig. 1A; Fig. S2). All of these genes are expressed at approximately equal mRNA levels (though at a lesser expression level than GAPDH), while an additional member of the RGS-R4 subfamily, RGS16, is not expressed in C3H10T1/2 cells (data not shown). Therefore, the necessary signaling components are present to assess the role of RGS5 in regulating the Shh signaling cascade. Following over-expression of RGS5 in C3H10T1/2 cells, Shh-reporter expression is inhibited. Specifically, Ptc1 and Ptc2 expression is inhibited by 28% and 35%, respectively (Fig. 1B and S3A). Furthermore, we confirm that RGS5 is over-expressed at both the mRNA and protein level (insets i and ii of Fig. 1B, respectively; see also Fig. S3C&D).

We extended our analysis to determine whether RGS5 over-expression had a functional consequence on the Shh signaling pathway, beyond simply inhibiting expression of individual components of the Shh cascade (Ptc1 and Ptc2; Fig. 1B). A classic functional pathway used to evaluate novel agonists and antagonists of the Shh pathway is osteogenic development [43], [58], [59]. Specifically, Shh stimulates the mRNA expression osteogenesis markers in C3H10T1/2 cells [58], and we hypothesized that over-expression of RGS5 would result in the inhibition of Shh-mediated induction of individual markers of osteogenic development. As shown in Fig. 1C and 1Di, hRGS5 remains over-expressed in transfected C3H10T1/2 cells following 21 days of culture, at least as measured by mRNA expression. Furthermore, we demonstrate that SAG, a small molecule agonist of the Hh pathway through the direct binding of Smo [60], also activates bone sialoprotein (Bsp) and osterix (Osx) similar to Shh stimulation [58], whereas collagen 1, type 1, alpha 1 (Col1α1) and Runx2 are not induced by SAG (Fig. S4). Importantly, in the presence of over-expressed hRGS5, expression of both Bsp and Osx is inhibited by ∼60% (Fig. 1D). Taken together, these results suggest that RGS5 functions downstream of Smo to regulate the Shh-mediated signal cascade.

### Shh Stimulation Inhibits RGS5 Message Expression

**Figure 2:**
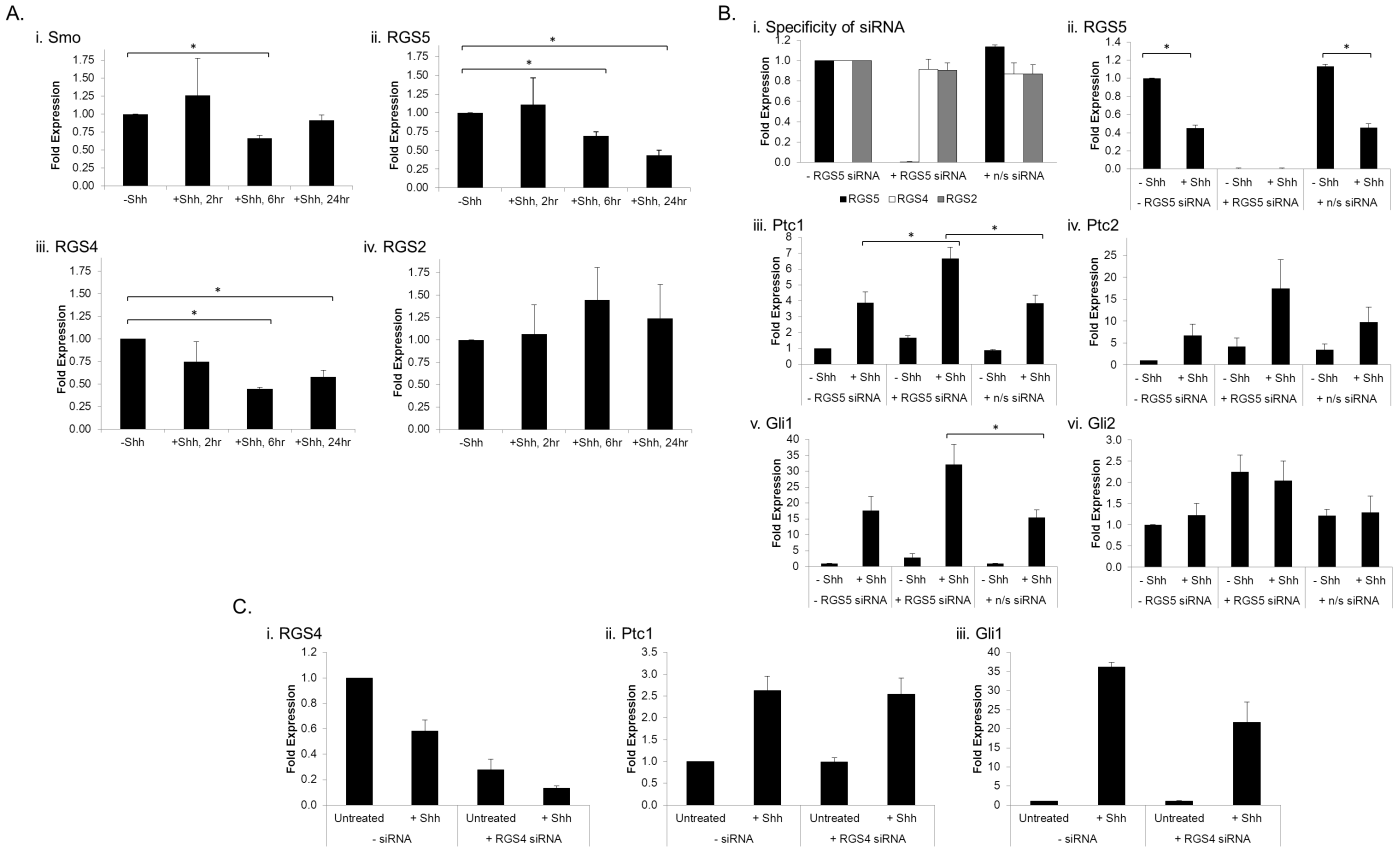
Direct effect of RGS5 on Shh-mediated reporter expression. (**A**) RGS5 and RGS4 are inhibited by Shh stimulation. C3H10T1/2 cells were stimulated with Shh (1 µg/mL) for 2 hours, 6 hours, and 24 hours. Relative expression of (i) Smo, (ii) RGS5, (iii) RGS4, and (iv) RGS2 was determined by qPCR, and normalized to the expression of each gene in the absence of Shh (untreated cells). n = 3–7; error bar = SEM; *p<0.05 by t-test. (**B**) Knock-down of RGS5 activates Shh-mediated reporter expression. The expression of RGS5 was knocked-down following transfection with gene-specific siRNA in C3H10T1/2 cells (i), whereas RGS4 and RGS2 expression is not affected by the RGS5 siRNA. Cells were treated with Shh (1 µg/mL) for 24 hours, and RNA was isolated. The relative expression of (ii) RGS5, (iii) Ptc1, (iv) Ptc2, (v) Gli1, and (vi) Gli2 was assessed by qPCR. Expression is corrected for GAPDH and normalized to the expression of each gene in the absence of siRNA transfection and in the absence of Shh stimulation (-Shh, -RGS5 siRNA). n = 4–5; error bars = SEM; *p<0.05 by t-test. (**C**) Knock-down of RGS4 does not potentiate Shh-mediated expression of reporter genes. The expression of RGS4 was knocked-down following transfection with gene-specific siRNA in C3H10T1/2 cells. Cells were treated with Shh (1 µg/mL) for 24 hours, and RNA was isolated. The relative expression of RGS4 (i), Ptc1 (ii) and Gli1 (iii) was assessed by qPCR. Expression is corrected for GAPDH and normalized to the expression of each gene in the absence of Shh stimulation (untreated, -siRNA). n = 3–5; error bars = SD.

To investigate whether Shh directly regulates the expression of members of the GPCR-mediated signaling complex, the mRNA expression of Smo, RGS5, RGS4, and RGS2 was determined following Shh treatment in C3H10T1/2 cells. Smo expression is transiently, but significantly, inhibited following 6 hours of Shh stimulation, but is not significantly different from basal expression following 24 hours of stimulation (Fig. 2A, i). Conversely, Shh stimulation for both 6 hours and 24 hours did affect expression of multiple members of the RGS-R4 subfamily. Specifically, RGS5 expression was down-regulated by approximately 60% (Fig. 2A, ii), and RGS4 expression was down-regulated by approximately 40% (Fig. 2A, iii) following 24 hours of Shh treatment. Conversely, Shh stimulation had no effect upon RGS2 expression (Fig. 2A, iv) at any of the time-points assayed. The Shh-dependent down-regulation of RGS5 and RGS4 implicates a feed-forward mechanism by which Shh actively down-regulates its repressor to augment signaling. Given the fact that RGS5 has been implicated as a biomarker in multiple cancers [61]–[65] and is expressed in pericytes [53]–[55], we focused on the effect of RGS5 upon Shh-mediated signaling.

### Knock-down of RGS5 Expression Activates Shh Reporter Expression

To determine the effect of RGS5 repression on Shh-mediated signaling, RGS5 expression was silenced by gene-specific siRNA. To confirm the specificity of the siRNA, we demonstrate that RGS5 expression is specifically knocked-down, whereas the siRNA failed to modulate expression of either RGS4 or RGS2 (Fig. 2B, i), two closely related members of the RGS-R4 subfamily of RGS proteins. Additionally, in agreement with the results in Fig. 2A, Shh treatment for 24 hours resulted in the inhibition of RGS5 expression (Fig. 2B, ii), both in the absence of gene-specific siRNA (left) and in the presence of non-specific siRNA (right).

As shown in Fig. 2B iii–vi, in the absence of gene-specific siRNA or in the presence of non-specific siRNA, Shh stimulation induced the expression of multiple Shh reporter genes: Ptc1 (iii), Ptc2 (iv), and Gli1 (v). However, Gli2 expression was not affected by Shh stimulation in C3H10T1/2 cells (Fig. 2B, vi), implying Gli2 expression might not be regulated in response to Shh in C3H10T1/2 cells. Importantly, when RGS5 expression was knocked-down by gene-specific siRNA, the expression of Ptc1, Ptc2, and Gli1 was induced approximately 2-fold (Fig. 2B), relative to expression of these Shh reporter genes in the absence of RGS5 siRNA. Similar effects were observed with an independent siRNA directed at RGS5 and in response to SAG-mediated activation of the Shh signaling cascade (Fig. S5). Therefore, in the absence of RGS5, the Shh-mediated induction of reporter gene expression is further potentiated.

In addition to RGS5, the expression of RGS4 is also inhibited by Shh stimulation (Fig. 2A, iii). To determine the effect of RGS4 expression on Shh-mediated signaling, RGS4 expression was silenced by gene specific siRNA (Fig. 2Ci). Unlike RGS5 knockdown, when RGS4 is inhibited by siRNA transfection, both Shh (Fig. 2Cii–iii) and the smoothened agonist SAG (Fig. S6) failed to further induce the expression of either Ptc1 or Gli1 (Fig. 2C; compare –*siRNA, +Shh* to *+RGS4 siRNA, +Shh*). Similar to RGS5, we over-expressed hRGS4 in C3H10T1/2 cells and demonstrated Shh-reporter gene expression is inhibited: Ptc1 by 70%, Ptc2 by 90%, and Gli1 by 95% (Fig. S7). Therefore, while expression of both RGS5 and RGS4 are inhibited by Shh stimulation and over-expression of both RGS5 and RGS4 inhibited Shh reporter expression, only the knock-down of RGS5 further potentiates the SAG- or Shh-mediated induction of Shh reporter gene expression. This data suggests that over-expression alone may result in non-specific effects, given the sensitivity of GPCR signaling to regulation by RGS proteins. However, given the correlated and antagonistic effects of both over-expression and knock-down of RGS5 expression, we are confident the inhibition of Smo-mediated signaling is unique and specific to RGS5 C3H10T1/2 cells.

### RGS5 is Present in the Primary Cilia

**Figure 3:**
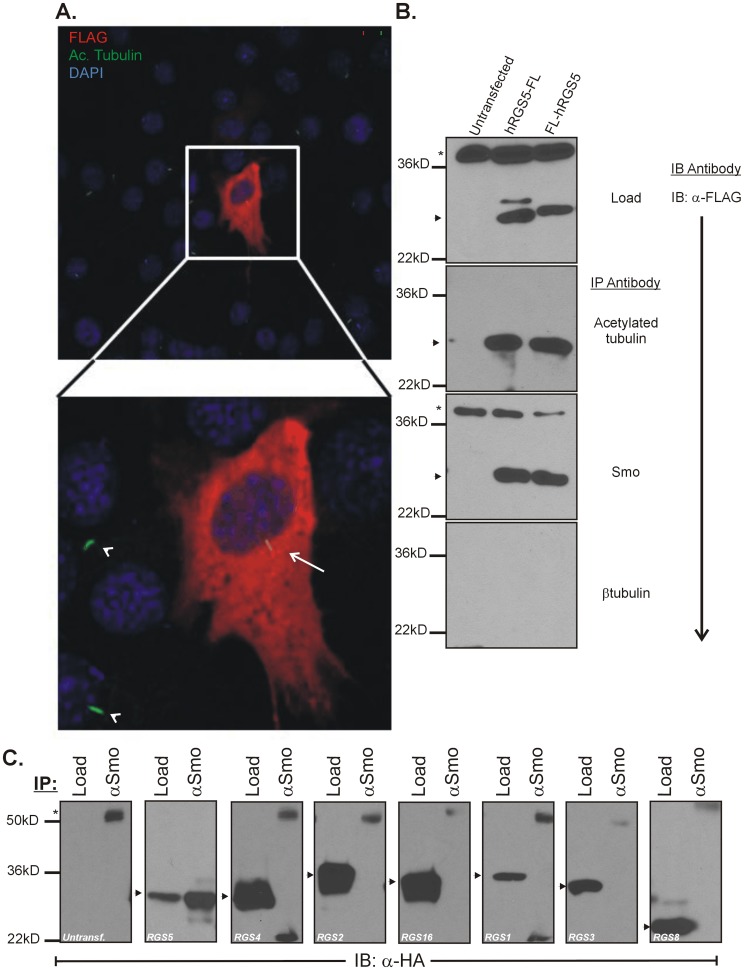
RGS5 is present in the primary cilia in C3H10T1/2 cells and interacts with acetylated tubulin and Smo. (**A**) C3H10T1/2 cells transfected with FL-hRGS5 were visualized by immunoflurescence. Cells were fixed and stained with α-acetylated tubulin (green), α-Flag (red), and nuclei are identified by DAPI staining (blue). Shown are primary cilia located on both transfected (arrow) and untransfected (arrowhead) cells. (**B**) RGS5 interacts with acetylated tubulin and Smo in the primary cilia of C3H10T1/2 cells. Cells were transfected with either FL-hRGS5 or hRGS5-FL. Protein complexes involving RGS5 were isolated by immunoprecipitation and analyzed by SDS/PAGE. Shown are positive interactions between RGS5 and acetylated tubulin and RGS5 and Smo. As a negative control, the β-tubulin antibody failed to immunoprecipitate RGS5 in transfected C3H10T1/2 cells. (**C**) The interaction between RGS5 and Smo is specific to RGS5 within the RGS-R4 subfamily of RGS proteins. Cells were transfected with HA-hRGS1, -hRGS2, -hRGS3, -hRGS4, -hRGS5, hRGS8, and -hRGS16. Protein complexes involving RGS proteins were isolated by immunoprecipitation with the α-Smo antibody and analyzed by SDS/PAGE and immunoblotting with the α-HA antibody. A positive interaction is shown between RGS5 and Smo, while RGS1, RGS2, RGS3, RGS4, RGS8, and RGS16 failed to interact with Smo. * = non-specific protein band; arrow head = FL-hRGS5 protein band.

Many studies have elucidated the requirement for the localization of components of the canonical Shh signaling cascade to the primary cilia [37], [39], [66]–[68]. Therefore, C3H10T1/2 cells were transfected with FL-RGS5 and analyzed for cellular localization by immunoflurescence. As shown in Fig. 3A, cilia are observed on cells transfected by FL-RGS5, as indicated by positive staining with the α-acetylated tubulin antibody. This implies that cilia disassembly does not occur when RGS5 is over-expressed, a potential mechanism for the repressive effect of over-expressed RGS5 [69]. However, due to the high expression levels of RGS5 in transfected cells, we found it difficult to confirm RGS5 was present in the primary cilia by either immunoflurescence (Fig. 3A) or confocal microscopy (data not shown). Therefore, we attempted to confirm localization of RGS5 to the primary cilia by co-immunoprecipitation (Co-IP).

C3H10T1/2 cells were transfected with FLAG epitope-tagged hRGS5 (either hRGS5-FL or FL-hRGS5; FLAG epitope fused to the C- or N-terminus, respectively). Potential interactions between RGS5 and components of the primary cilia were analyzed by Co-IP and SDS-PAGE. We demonstrate in Fig. 3B that when RGS5 is over-expressed in C3H10T1/2 cells, RGS5 is capable of interacting with both acetylated tubulin and Smo, while RGS5 does not non-specifically interact with β-tubulin. Under the conditions used, acetylated tubulin (green) is observed exclusively in the primary cilia of C3H10T1/2 cells (Fig. 3A, lower panel, arrowheads). Furthermore, as a control, we demonstrated that the antibodies to acetylated tubulin and Smo are specific, as Co-IP with α-mouse IgG or α-rabbit IgG failed to immunoprecipitate FL-hRGS5 from transfected cells (Fig. S8). Therefore, not only does RGS5 regulate the Shh-mediated signaling cascade, but it is present in the primary cilia and interacts with both acetylated tubulin and the GPCR responsible for Shh-mediated signaling (Smo).

To test whether the co-localization of Smo and RGS5 to the primary cilia is specific to RGS5, we analyzed the localization of multiple additional members of the RGS-R4 subfamily by Co-IP. As shown in Fig. 3C, while RGS5 was Co-IPed with Smo, RGS1, RGS2, RGS3, RGS4, RGS8, and RGS16 failed to interact with Smo in C3H10T1/2 cells. This implies a specific interaction between Smo and RGS5 in primary cilia, at least in relation to the cell type and RGS protein assayed.

## Discussion

We provide evidence that RGS proteins regulate canonical Hh signaling at the level of Smo-mediated G-protein coupling to downstream effector pathways in mammalian cells. RGS proteins accelerate GTP hydrolysis by Gα proteins and thereby inhibit GPCR-mediated signaling [25]. We found that over-expression of RGS5 inhibits gene expression downstream of Smo in C3H10T1/2 cells (Fig. 1B) and functionally inhibits Hh-dependent osteogenic development (Fig. 1D). Conversely, loss of RGS5 function led to increased levels of Shh-stimulated gene expression (Fig. 2B). Moreover, RGS5 was found to be present with acetylated tubulin in the primary cilia (Fig. 3A) and could be Co-IPed in a complex with Smo and acetylated tubulin (Fig. 3B). Taken together, these results demonstrate that RGS5 functions as an inhibitor of Hh signaling downstream of Smo. We propose the interaction between Smo, Gα subunits, and RGS proteins may provide novel targets for the control of Hh-mediated signaling in human disease.

**Figure 4:**
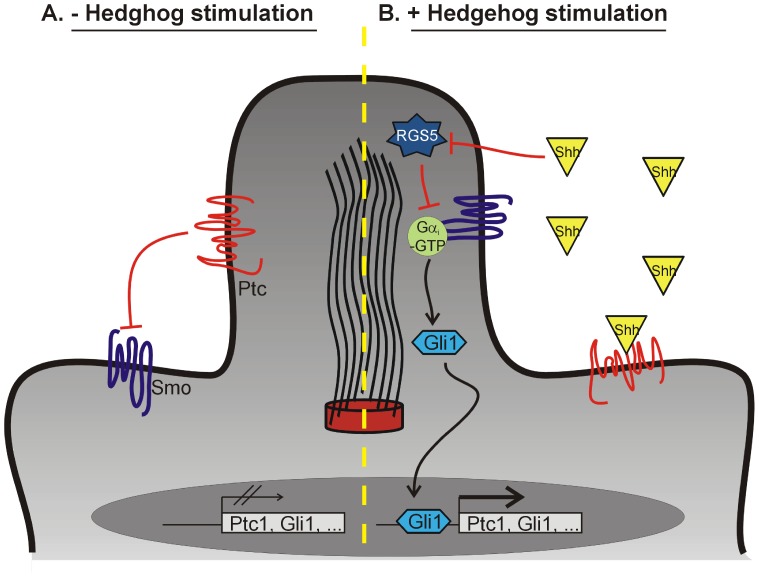
The hedgehog-mediated signaling mechanism in the absence (A) and presence (B) of Shh. RGS5 inhibits Shh-mediated signaling. RGS5 functions to inhibit signaling down-stream of Smo by hydrolyzing Gα_i_-GTP. In the absence of Shh, RGS5 inhibits Smo-dependent signaling by inactivating Gα_i_ and blocking the expression of the Gli transcription factors and Ptc co-receptors. In the presence of Shh, RGS5 expression is repressed, leading to the potentiation of the activation of Gli and Ptc expression.

Smo is an integral membrane protein with significant structural homology to GPCRs [11]–[13]. In the unstimulated state, Ptc proteins inhibit Smo signaling, presumably by preventing Smo localization to the primary cilia ([36]; Fig. 4A). However, upon binding Hh proteins (Shh, indian hedgehog (Ihh), or desert hedgehog (Dhh)), Ptc leaves and Smo enters the primary cilia, where it resides in close proximity to other components of the Shh signaling complex: the Gli transcription factors and the large G proteins (Fig. 4B) [1]–[3], [28], [70], [71]. Multiple recent studies have characterized the interactions between Smo and members of the large G protein family. In Drosophila, Ogden *et al* demonstrated that Smo signals through Gα_i_ [38]. In mammalian cells, Riobo *et al* demonstrated that Smo interacts with Gα_i_ [37], and interactions between Smo and Gα_i_ have been implicated in the control of both cell migration [68] and proliferation [39]. Interestingly, Kasai *et al* demonstrated that Smo may interact with Gα_12/13_ in neuroblastoma cells [72], however, Douglas *et al* recently determined that the activation of the Gli transcription factors by Gα_13_ does not occur in every cell type and is independent of Smo [66]. A similar argument of cell-specific activity of Gα_i_ proteins was proposed by Hammerschmidt and McMahon, who demonstrated that blocking Gα_i_-mediated signaling with pertussis toxin affected some, but not all Hh-dependent developmental processes in zebrafish [51]. Finally, a recent study by Manning and colleagues demonstrated that, at least *in vitro*, Smo is capable of activating Gα_i_ with an equivalent activity as the serotonin receptor [14]. We hypothesized that RGS5 functions to regulate signaling through Smo, given that fact that RGS proteins catalyze the hydrolysis of Gα-GTP to Gα-GDP, and that RGS5 specifically interacts with Gα_i_ and Gα_q_ [40]. Furthermore, our data suggests RGS5 regulates canonical Hh signaling [68], [73], since we observe an RGS5-dependent effect upon Hh target gene transcription and we demonstrate a physical interaction between RGS5, Smo, and acetylated tubulin in the primary cilia. Our study therefore identifies RGS5 as a novel regulator of the Shh signaling cascade (Fig. 4B).

Many studies have implicated both Shh and RGS5 in the control of vascular development and remodeling in response to injury. In a model of hindlimb ischemia, the Shh signaling cascade is up-regulated in the interstitum, and blocking this pathway inhibits collateral vessel formation, whereas up-regulating the pathway enhances recovery from hindlimb ischemia [74]–[76]. Shh is also a regulator of coronary artery development [77]–[79], as well as carotid artery intimal hyperplasia [80], [81]. We are intrigued by the juxtaposition of expression domains for RGS5 and the targets of Shh signaling. For example, RGS5 is robustly expressed in pericytes [82], [83] and in arterial vascular smooth muscle cells [84]–[87]. Conversely, despite expression of Shh protein in the border zone between the media and adventitia [8], expression of Shh signaling reporters Ptc1, Ptc2, and Gli1 is restricted to the vascular adventitia [8], [88], [89]. We hypothesize that in the uninjured vessel wall, RGS5 expression in medial smooth muscle cells restricts expression of Shh and reporter genes to the adjacent adventitia. However, following vascular injury, RGS5 expression is inhibited [87], potentially as a result of local increases in PDGF-BB [41], thereby allowing the Shh signaling domain to expand to the vascular media and neointima [81].

In addition to vascular development and remodeling, Shh-mediated signaling and RGS proteins have been implicated in the development of multiple cancers [90]. Of interest to our studies, multiple members of the RGS-R4 subfamily have been associated with cancers that exhibit aberrant Shh signaling. For example, increased Shh signaling has been correlated with poor prognosis in ovarian cancer [61], breast cancer [91]–[93], medulloblastoma [90], and hepatocellular carcinoma [94]–[97]. Misexpression of RGS2 [61], RGS4 [61], and RGS5 [62]–[65] has also been reported in a number of these cancers. Recently, RGS proteins themselves have been pharmacologically targeted (reviewed in Kimple *et al* [98]). For example, chemical screens have identified novel inhibitors of RGS4 [99]–[101], RGS8 [102], and RGS20 [103]. Therefore, a potential treatment option for cancers in which the Shh signaling pathway is aberrantly activated would be to promote RGS5 expression or prolong its activity at the tumor site. We hypothesize this would sensitize the cancer to treatment with hedgehog antagonists, thereby requiring a lower drug treatment which might prevent undesirable off-target effects of inhibiting the hedgehog pathway [78], [104]–[106].

Our data raise several important questions going forward. For example, (i) do RGS proteins regulate Smo-mediated signaling in cell types other than C3H10T1/2; (ii) will RGS5-mediated inhibition of Shh target gene expression be followed by corresponding effects on other biological endpoints beyond simple gene expression; (iii) is trafficking of RGS proteins to the primary cilia regulated by Shh signaling and can a specific domain of the RGS protein be identified that mediates such trafficking; (iv) can RGS proteins also inhibit non-canonical forms of Shh signaling [68]; (v) does down-regulation of RGS protein expression by Shh lead to enhanced signaling by other GPCRs that couple to Gα_i_-dependent pathways; (vi) does RGS5 expression affect the Gli3 repressor/activator ratio in cells; and (vii) do apparently normal appearing RGS5-null mice [107]–[109] exhibit Shh signaling defects *in vivo* when carefully examined following exposure to injury or disease-causing stimuli?

In summary, our study presents data demonstrating RGS5 is a novel regulator of the Shh signaling cascade. In the context of the recent studies describing interactions between the heterotrimeric G proteins and Smo, it is not surprising that RGS proteins participate in the control of Shh-mediated signaling, and we propose the interaction between Shh signaling and RGS proteins may represent novel targets in the control of both cancer and vascular remodeling and disease.

## Supporting Information

Figure S1 **Effect of PTX on SAG-mediated Ptc1 and Gli1 expression.** Hedgehog-mediated gene expression is sensitive to pertussis toxin (PTX) in C3H10T1/2 cells, and therefore signals through Gαi. C3H10T1/2 cells were stimulated with SAG (24 hrs; 100 nM) in the presence of PTX (24 hrs; 100 ng/mL; List Biological Laboratories Inc.) or vehicle (24 hrs; 0.1% BSA in PBS). RNA was isolated and gene expression of Ptc1 **(A)** and Gli1 **(B)** was determined as described in *Methods and Materials.* Gene expression was corrected for GAPDH expression and normalized to expression in the presence of SAG: Fold  = 2−ΔΔCt. (n  = 3; error bar = SEM; * = p<0.05 by t-test).
(TIF)

Figure S2 **Relative Expression in C3H10T1/2 cells.** Quantification of relative gene expression in C3H10T1/2 cells. The basal expression level of various members of the RGS-R4 subfamily (RGS5, RGS2, and RGS4) and members of the Shh signaling cascade (Smo, Ptc1, Ptc2, Gli1 and Gli2) in C3H10T1/2 cells. Expression of the indicated genes was determined by qPCR. Data was corrected for GAPDH expression and normalized to expression of each gene in a single sample. (n = 3–7; error bars = SEM). This data is re-plotted from frigure 1A.
(TIF)

Figure S3 **Confirmation of hRGS5 over-expression by qPCR, RT-PCR, and immunoblot.** Representative confirmation of hRGS5 expression when Shh/SAG-mediated gene expression is inhibited. C3H10T1/2 cells were cultured in the absence or presence of SAG (100 nM) and the presence or absence of transiently over-expressed FL-hRGS5 for 24 hrs. **(A)** RNA was isolated and the expression of Shh reporter genes (Ptc1, Ptc2, Gli1) was determined by qPCR as described. Expression values were corrected for GAPDH expression and normalized to the condition ‘+SAG, -hRGS5’ for each gene assayed (Fold Expression = 2−ΔΔCt). **(B)** The expression of transiently over-expressed hRGS5 was quantitated by qPCR as above. **(C)** The expression of transiently over-expressed RGS5 was confirmed by traditional RT-PCR. Shown is the product following 30 amplification cycles and separation on a 1% SDS-PAGE gel. **(D)** Protein expression for FL-hRGS5 was confirmed by immunonblot with the α-FLAG antibody. Whole cell extract from treated and/or transfected cells was separated by SDS-PAGE, probed with the α-FLAG antibody and visualized by ECL detection, as described.
(TIF)

Figure S4 **RGS5-mediated inhibition of SAG-induced osteogenesis.** RGS5 over-expression inhibits SAG-induced osteogenesis in C3H10T1/2 cells. The expression of multiple markers of osteogenesis was assayed in the presence or absence of over-expressed RGS5 and the presence or absence of SAG (SAG *was used because it is more stable than the recombinant Shh protein in long-term culture).* Specifically, C3H10T1/2 cells were cultured for 21 days after being transiently transfected with FL- hRGS5 and treated with SAG (100 nM). Media was changed every 3–4 days following transfection and SAG treatment. RNA was isolated and gene expression of **(A)** RGS5, **(B)** collagen, type 1, alpha 1 (Col1α1), bone sialoprotein (Bsp), osterix (Osx), and related transcription factor 2 (Runx2) was determined. Expression of both Bsp and Osx was induced by SAG treatment, and expression of both genes is inhibited by the over- expression of RGS5. Conversely, neither Col1α1 or Runx2 was significantly stimulated by 21 days of SAG treatment. Gene expression was corrected for GAPDH expression and normalized to the expression of each gene in the absence of RGS5 over-expression and in the absence of SAG treatment (white bar). (n = 3; error bar = SEM; * = p<0.05).
(TIF)

Figure S5 **Effect of multiple RGS5 siRNAs on SAG- mediated expression of Ptc1 and Gli1.** Multiple siRNAs targeting murine RGS5 have similar effects upon Hedgehog-mediated gene expression. C3H10T1/2 cells were transiently transfected with 2 independent siRNAs targeting murine RGS5, as described in *Methods and Materials.* siRNA (1) is described in the manuscript, while siRNA (2) has the following sequence (5′- GGUGAACAUUGACCACUUCACUAAA-3′; Invitrogen). Cells were stimulated with SAG (24 hrs; 100 nM) either in the presence or absence of the individual siRNAs. RNA as isolated and gene expression of RGS5 **(A),** Ptc1 **(B),** and Gli1 **(C)** was determined. Both siRNAs inhibited RGS5 expression **(A)** and expression of both Ptc1 **(B)** and Gli1 **(C)** was potentiated in the absence of RGS5. Gene expression was corrected for GAPDH expression and normalized to expression in the absence of siRNA. (n = 2; error bar = SD).
(TIF)

Figure S6 **Effect of RGS4 knockdown on SAG-mediated induction of Shh reporter gene expression.** Knock-down of RGS4 does not activate SAG-mediated expression of Shh reporter genes. The expression of RGS4 was knocked-down following transfection with a gene-specific siRNA in C3H10T1/2 cells. Cells were treated with SAG (100 nM) for 24 hrs, and RNA was isolated. The relative expression of Ptc1 **(i)** and Gli1 **(ii)** was assed by qPCR. Expression is corrected for GAPDH and normalized to the expression of each gene in the absence of SAG stimulation (untreated, −siRNA). (n = 3–5; error bar = SEM; p<0.05 by t-test). Importantly, note that knock-down of RGS5 potentiated SAG-mediated up-regulation of Ptc1 and Gli1, whereas knock-down of RGS4 failed to further up-regulate the expression of these genes.
(TIF)

Figure S7 **Effect of RGS4 over-expression on Shh- mediated gene expression.** Over-expressed RGS4 inhibits Shh-mediated reporter expression. RGS4 (HA-hRGS4) was over-expressed in C3H10T1/2 cells by transient transfection and treated with SAG (100 nM) for 24 hours. The effect of RGS4 over-expression on Ptc1 and Gli1 was assessed by qPCR. Expression was corrected for GAPHD expression and normalized to the expression of each gene following 24 hr of SAG treatment, but in the absence of RGS4 over- expression (white bar). (n = 2; error bar = SD).
(TIF)

Figure S8 **Assay for Specific Interaction between RGS5:Ac-Tubulin and RGS5:Smo.** RGS5 specifically interacts with acetylated tubulin and Smoothened. C3H10T1/2 cells were transiently transfected with FL-RGS5. Protein complexes were immunoprecipitated (IPed) with antibodies to acetylated tubulin or mouse IgG control and Smo or rabbit IgG control and analyzed by SDS/PAGE. Samples were immunobloted with the α-Flag antibody, demonstrating the presence of RGS5 in the IPed protein complexes. Shown are positive and specific interactions between RGS5 and acetylated tubulin and RGS5 and Smo, but not between RGS5 and the IgG controls, respectively (arrow head = FL-hRGS5).
(TIF)
