# Supplementary figures and images for: Regulator of G-Protein Signaling – 5 (RGS5) Is a Novel Repressor of Hedgehog Signaling
Source: PLoS One. 2013 Apr 18;8(4):e61421. doi: 10.1371/journal.pone.0061421 (PMC3630190; doi:10.1371/journal.pone.0061421)

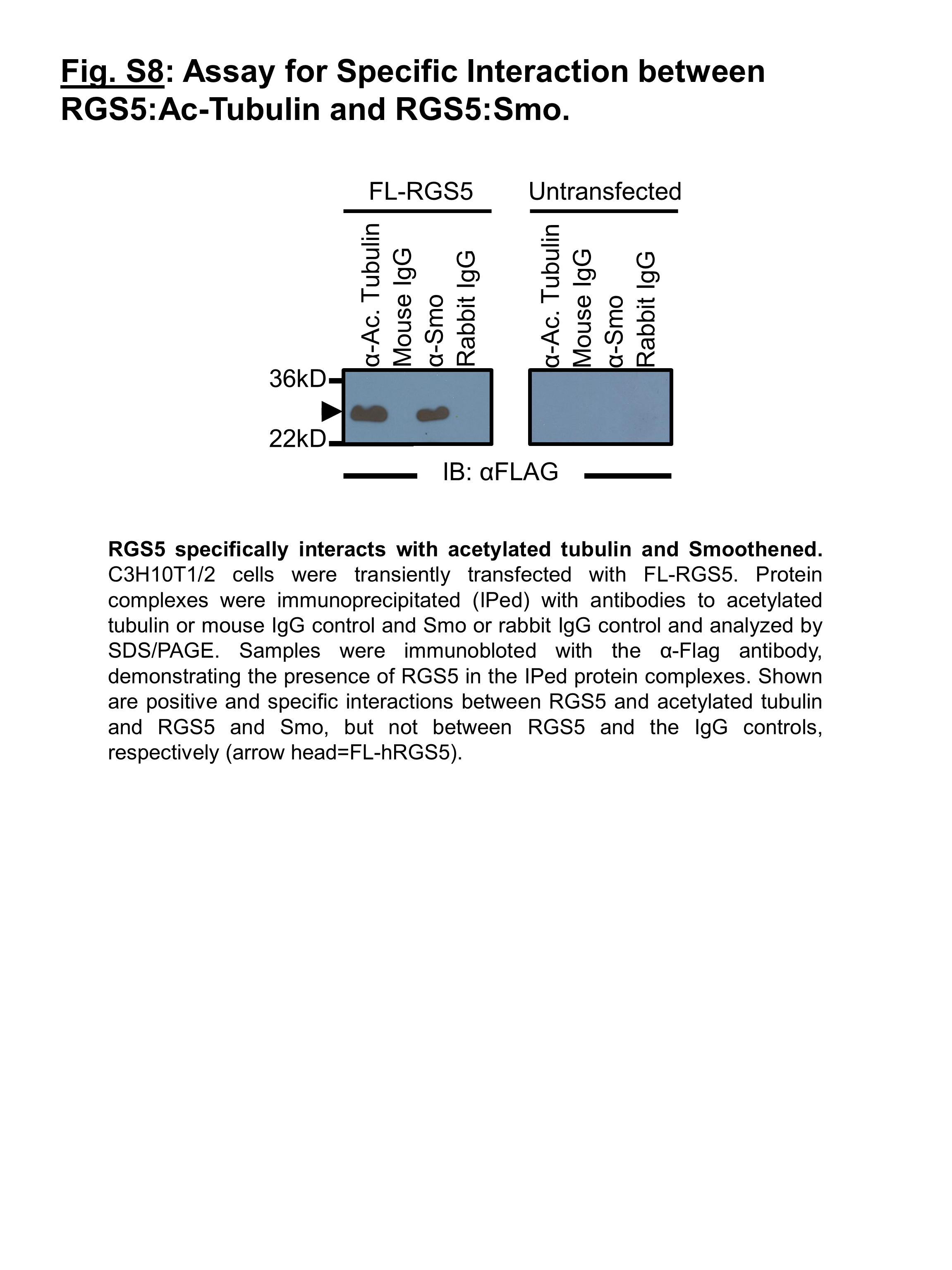

Supplement: Figure S8 — Assay for Specific Interaction between RGS5:Ac-Tubulin and RGS5:Smo. RGS5 specifically interacts with acetylated tubulin and Smoothened. C3H10T1/2 cells were transiently transfected with FL-RGS5. Protein complexes were immunoprecipitated (IPed) with antibodies to acetylated tubulin or mouse IgG control and Smo or rabbit IgG control and analyzed by SDS/PAGE. Samples were immunobloted with the α-Flag antibody, demonstrating the presence of RGS5 in the IPed protein complexes. Shown are positive and specific interactions between RGS5 and acetylated tubulin and RGS5 and Smo, but not between RGS5 and the IgG controls, respectively (arrow head = FL-hRGS5). (TIF) [file pone.0061421.s008.tif]
